# Interaction between Social/Psychosocial Factors and Genetic Variants on Body Mass Index: A Gene-Environment Interaction Analysis in a Longitudinal Setting

**DOI:** 10.3390/ijerph14101153

**Published:** 2017-09-29

**Authors:** Wei Zhao, Erin B. Ware, Zihuai He, Sharon L. R. Kardia, Jessica D. Faul, Jennifer A. Smith

**Affiliations:** 1 Department of Epidemiology, School of Public Health, University of Michigan, Ann Arbor, MI 48109, USA; skardia@umich.edu (S.L.R.K.); smjenn@umich.edu (J.A.S.); 2 Survey Research Center, Institute for Social Research, University of Michigan, Ann Arbor, MI 48104, USA; ebakshis@umich.edu (E.B.W.); jfaul@umich.edu (J.D.F.); 3 Department of Biostatistics, Columbia University, New York, NY 10032, USA; zihuai@umich.edu

**Keywords:** gene-environment interaction, body mass index, social/psychosocial factors, longitudinal analysis, LGEWIS

## Abstract

Obesity, which develops over time, is one of the leading causes of chronic diseases such as cardiovascular disease. However, hundreds of BMI (body mass index)-associated genetic loci identified through large-scale genome-wide association studies (GWAS) only explain about 2.7% of BMI variation. Most common human traits are believed to be influenced by both genetic and environmental factors. Past studies suggest a variety of environmental features that are associated with obesity, including socioeconomic status and psychosocial factors. This study combines both gene/regions and environmental factors to explore whether social/psychosocial factors (childhood and adult socioeconomic status, social support, anger, chronic burden, stressful life events, and depressive symptoms) modify the effect of sets of genetic variants on BMI in European American and African American participants in the Health and Retirement Study (HRS). In order to incorporate longitudinal phenotype data collected in the HRS and investigate entire sets of single nucleotide polymorphisms (SNPs) within gene/region simultaneously, we applied a novel set-based test for gene-environment interaction in longitudinal studies (LGEWIS). Childhood socioeconomic status (parental education) was found to modify the genetic effect in the gene/region around SNP rs9540493 on BMI in European Americans in the HRS. The most significant SNP (rs9540488) by childhood socioeconomic status interaction within the rs9540493 gene/region was suggestively replicated in the Multi-Ethnic Study of Atherosclerosis (MESA) (*p* = 0.07).

## 1. Introduction

Obesity is a leading cause of age-related chronic diseases including cardiovascular disease, type 2 diabetes, and hyperlipidemia [1,2]. There are persistent and pronounced differences in body mass index (BMI), an indicator of obesity, across ethnic ancestries. Understanding the factors that contribute to BMI is critical for developing effective BMI treatment strategies that may lead to reductions in obesity and ultimately a decrease in the incidence of obesity-related chronic disease.

Recent studies suggest that genetic background may play an important role in the development of obesity. Multiple BMI-associated genetic loci have been identified through large-scale genome-wide association studies (GWAS) [3,4,5]. While estimates of the heritability of BMI range from 40% to 70% [6,7], these genetic variants only explain about 2.7% of BMI variation in total, leaving a large amount of heritability unexplained [5]. Most common human traits are believed to be influenced by both genetic and environmental factors. Past studies suggest a variety of environmental features that are associated with obesity, including socioeconomic status and psychosocial factors [8,9,10]. There is also evidence that genetic factors related to BMI and adiposity are socially moderated [11,12]. The large amount of missing heritability could be due to gene-environment interaction [11,13,14,15].

This study combines both genetic and environmental risk factors to explore whether social/psychosocial environments modify the effect of sets of genetic variants on BMI. We examined the interaction between BMI risk loci and multiple social/psychosocial factors on BMI in European American (EA) and African American (AA) participants in the Health and Retirement Study (HRS). Traditionally, genetic variants are tested one at a time, a method which suffers from less power due to extensive multiple testing. Set-based tests represent a way to aggregate many variants in a gene/region to simultaneously test their effect on a phenotype, thereby reducing the number of tests and bringing the level of inference to that of the gene. This gene-level inference is an important property when comparing genetic determinants of disease across ethnicities due to the inherent population stratification and admixture that is present within ethnic groups. In order to effectively incorporate the rich longitudinal phenotype data collected in the HRS and reduce the multiple testing burden, we applied a novel set-based test for gene-environment interaction in longitudinal studies (LGEWIS) [16]. Following discovery analyses, we sought replication of significant findings in the Multi-Ethnic Study of Atherosclerosis (MESA). By incorporating both biological and social/psychosocial data, we seek to better understand the etiology of obesity. We hypothesize that there will be gene/regions associated with BMI in both EA and AA samples, while socioeconomic status, social support, anger, chronic burden, stressful life events, and depressive symptoms will modify the effects of genetic predictors on BMI over time.

## 2. Materials and Methods

### 2.1. Discovery Sample

The HRS is a nationally-representative longitudinal panel study of approximately 26,000 adults over the age of 50 years [17]. Core interview data is collected every two years using a mixed mode design, combining in-person and telephone interviews. In 2006, a random one-half of households with at least one living participant were pre-selected to complete an enhanced face-to-face (EFTF) interview, which included a set of physical performance tests, anthropometric measurements, blood and saliva samples, and a psychosocial self-administered questionnaire in addition to the core HRS interview. The sample was selected at the household level. In 2008, an EFTF interview was conducted on the remaining half of the sample. Similarly, new cohort households for 2010 were randomly assigned into one of these two groups with half being asked to complete an EFTF interview in 2010 and the other half to complete an EFTF interview in 2012. EFTF interview data collected from 2008 to 2012 was included for analysis. Since new participants were recruited in 2010, some of the participants only had one EFTF interview. For each participant, the EFTF interview that was conducted first is referred to as Exam 1, and the EFTF interview that was conducted four years later is referred to as Exam 2 in these analyses.

### 2.2. Outcome and Adjustment Covariates

BMI (kg/m^2^), the primary outcome of interest in this analysis, is calculated from measures of height and weight collected during EFTF interviews. Outliers that were greater than 6 standard deviations from the mean BMI were excluded. Sex from the HRS core survey files, and age (years) for each exam available were included as covariates. The top four within-ethnicity genetic principal components were included in genetic models to account for population stratification.

### 2.3. Social/Psychosocial Measures

Two measures of socioeconomic status are incorporated as effect modifiers: childhood socioeconomic status (CSES, maximum parental educational attainment) and adult socioeconomic status (ASES, individual educational attainment). CSES was dichotomized as a high school degree or higher (for the highest educational attainment of either parent) as the reference category, while ASES was dichotomized with greater than 12 years of education (equivalent to some college education) as the reference category. Different cut-off points were used due to different educational opportunities for different generations (parents vs. participants).

Psychosocial measures included: trait anger (ANGERIN), state anger (ANGEROUT), chronic burden (BURDEN), stressful life events (SLE), positive and negative social support (PSS and NSS), and depressive symptoms (CESD).

The Spielberger Anger Expression Scale (STAX) measures anger along two dimensions: state anger and trait anger. Trait anger (ANGERIN) refers to a more stable predisposition to respond to a range of situations with an angry response, while state anger (ANGEROUT) represents a more temporary angry reaction usually expressed through behavior [18,19]. Each item was measured on a scale from 1 (“almost never”) to 4 (“almost always”), and anger scores were calculated as the average item response. Items were reverse coded as necessary and any participants with more than two missing responses for the trait anger battery (out of four) or more than three missing responses for the state anger battery (out of seven) was coded as missing.

Chronic burden (BURDEN) was assessed by eight items that asked the participants if they have current and ongoing problems that have lasted twelve months or longer and how upsetting it has been. The domains that were assessed included a health problem, physical or emotional problem, alcohol or drug use in family members, difficulties at work, financial strain, housing problems, a problem in a close relationship, and helping a sick, limited, or frail family member or friend [20,21,22]. Each item was measured on a scale from 1 (“No, didn’t happen”) to 4 (“Yes, very upsetting”). A composite score was created by summing the individual scores.

Stressful life events (SLE) were measured by six items in the questionnaire. The questionnaire asked the participant if he/she experienced any of the following stressful life events in the past five years: lost a job, was unemployed/looking for a job for more than three months, any member in the household was unemployed/looking for a job for longer than three months, moved to a worse residence or neighborhood, got robbed or had home burglarized, was the victim of fraud. Each item was coded as 1 (“Yes”) or 0 (“No”). A composite score was created by summing the individual scores [23].

Positive Social Support (PSS) was measured by three items in four different domains: (a) How much do they really understand the way you feel about things? (b) How much can you rely on them if you have a serious problem? And (c) How much can you open up to them if you need to talk about your worries? Negative Social Support (NSS) was measured by four items: (d) How often do they make too many demands on you? (e) How much do they criticize you? (f) How much do they let you down when you are counting on them? and (g) How much do they get on your nerves? Separate questions are asked for each of the four domains, including spouse/partner, children, family, and friends. All items were measured on a scale from 1 (“A lot”) to 4 (“Not at all”). All items were first reverse coded, and then an index of PSS and an index of NSS were created for each relationship domain by averaging the scores within each dimension (positive (a–c) and negative (d–g)). The final score was set to missing if there was more than one item with missing values for the positive social support scale, or more than two items with missing values for the negative social support scale [24,25]. The scores from the four relationship domains were averaged to get the final PSS and NSS score for each exam.

Depressive symptoms (CESD) were assessed by the eight items in the HRS depressive symptom questionnaire based on the Center for Epidemiologic Studies–Depression scale [26]. CESD variables were taken from the RAND HRS data file [27] which summarizes the eight HRS items. Each participant was asked the following questions with “yes” or “no” response options: (a) Much of the time during the past week, I felt depressed, (b) I felt everything I did was an effort, (c) My sleep was restless, (d) I was happy, (e) I felt lonely, (f) I enjoyed life, (g) I felt sad, and (h) I could not “get going”. The total number of “yes” responses to questions a, b, c, e, g, and h, and the “no” responses to questions d and f were summed to be the total depressive symptom score ranging from 0 to 8.

The details of the collection method of all the aforementioned social/psychosocial measures have been described previously by the HRS [28]. All psychosocial variables except CESD and SLE were dichotomized at the ethnicity-specific median (combining all measures over time). Both CESD and SLE were modeled as dichotomous, with a zero count as the reference category. The chronic burden questionnaire was not administered in 2008, which resulted in a smaller number of observations for this variable (BURDEN). In summary, all the social/psychosocial factors were dichotomized (0 and 1) with 1 representing the adverse social/psychosocial category.

### 2.4. Genotype Data

Salivary DNA was collected from HRS participants during EFTF interviews in 2006, 2008, and 2010. Genotyping was conducted by the Center for Inherited Disease Research (CIDR). Genotype data was obtained using the Illumina HumanOmni2.5 BeadChip, which measures 2.4 million single nucleotide polymorphisms (SNPs). Imputation to the 1000 Genomes Project cosmopolitan reference panel phase 1, version 3 (released on March 2012) was performed using SHAPEIT2 [29] and IMPUTE2 [30]. Details of the genotyping, quality control and imputation are described in the Supplementary Materials. For each ethnicity, genetic principal components were generated from common SNPs (MAF > 0.05) using Genome-wide Complex Trait Analysis (GCTA) [31] and were used as covariates to adjust for population stratification.

The gene/regions were selected from 97 index SNPs that have been previously identified to be associated with BMI (*p* < 5 × 10^−8^) in the largest replicated GWAS to date [5]. The BMI risk gene/regions were defined in the following way: if the index SNP from the GWAS was within the boundaries of a gene plus 5 kb buffer on either side (surrounding the gene), all SNPs within the gene were selected (54 genes). If the index SNP was not in a gene, all SNPs within 50 kb of the index SNP were selected (43 regions). All position information was based on genome assembly GRCh37, and gene positions were defined by GENCODE annotation version 19 [32]. If there were multiple transcripts for a gene, the most inclusive start and stop positions were used to define the gene. Imputed SNPs within the defined gene/region with good imputation quality (INFO > 0.5) and minor allele frequency (MAF) >0.01 were retained for analysis. The number of SNPs in these 97 gene/regions ranged from 38 to 7287 for the EA sample, and from 71 to 11,583 for the AA sample (Table S1).

All data was collected during the 2006–2012 interview waves. Participants with missing BMI or missing all of the social/psychosocial variables were removed. The final sample size is 7838 for EA and 1334 for AA. Among EA participants, 4571 subjects have measurements from two exams, while 3267 have only one measurement. Among the AA participants, 527 subjects have measurements from two exams, while 807 have one measurement.

### 2.5. Statistical Methods

The marginal effect of social/psychosocial factors on BMI was tested using generalized estimating equation (GEE) models in EA and AA separately. The models were adjusted for age, age^2^, and sex. Inverse variance-based fixed effect meta-analysis was used to assess the marginal effect of social/psychosocial factors on BMI in the combined sample (EA + AA). Bonferroni correction was applied to the nine tests (CSES, ASES, ANGERIN, ANGEROUT, BURDEN, SLE, PSS, NSS, CESD) conducted for each ethnic group.

Genetic association is often statistically assessed by fitting a regression model for each SNP independently. In a genome wide search, millions of tests for individual SNPs are conducted, and results are adjusted for multiple testing. This limits the statistical power of the tests. LGEWIS [16] is a GEE-based dispersion test specifically designed for longitudinal studies to test the joint effect of genetic variants or gene-environment interactions in a gene/region on phenotypic variation. Briefly, *Y_ij_* denotes the outcome variable for *i*-th subject at time *j*, *E_ij_* represents the environmental exposures for the *j*-th observation on the *i*-th subject measured at time *j*; *X_ij_* denotes covariates and are defined similarly as *E_ij_*. G*_i_*= (*G_i_*_1_*, G_i_*_2_*,·····, G_ip_*) represents the genotypes for the *p* variants within the gene/region for individual *i*. *G_i_* remains the same across different time points *j*. We are primarily interested in the statistical interaction between *E_ij_* and *G_i_* on outcome *Y_ij_*, adjusting for *X_ij_* and the main effect of *E_ij_* and *G_i_.* The statistical model is:

(1) $$Y_{ij}=\alpha^{\prime }X_{ij}+f(E_{ij})+\beta^{\prime }G_{i}+\gamma^{\prime }(E_{ij}*G_{i})+e_{ij}$$

where α = [α_1_*,…,*α*_m_*]′ is the vector of regression coefficients for the *m* covariates, and *f(E_ij_*) is the spline smoothing function to capture the possibly nonlinear main environmental effect. The literature [33,34,35] has shown that a mis-specified main effect of E can cause severe type I error inflation. It was theoretically proven that modeling the nonlinear main effect of E greatly helps in controlling type I error rate and does not substantially hurt power for tests of gene-environment interaction. *β* = [*β*_1_*,…,β_p_*]′ is the vector of regression coefficients for the *p* observed SNPs*,* and *γ*′ = [*γ*_1_*,…,γ_p_*]′ is the vector coefficient of the interaction terms for the environment and the *p* observed SNPs. In the analysis of the HRS, the number of variants in a gene can be large relative to the sample size. Directly fitting a high-dimensional main effect of G suffers from severe type I error inflation. Therefore, LGEWIS uses a new weighted PCA (principal component analysis) approach for the main effect of G. Under the scenario in which the number of SNPs is small relative to the sample size, the approach reverts to the standard method of adjusting for all SNPs. The goal is to evaluate the H_0_: *γ*_1_ *= γ*_2_ *= … = γ_p_* = 0, and an aggregated score statistic is used to test the overall deviation of *γ* from 0. We applied a similar dispersion test to evaluate the marginal genetic effect by testing H_0_: *β* = 0 in model

(2) $$Y_{ij}=\alpha^{\prime }X_{ij}+\beta^{\prime }G_{i}+e_{ij}$$

In this study, LGEWIS was used to test the association between BMI and the SNP set within each gene/region after adjusting for age, age^2^, sex, and the top four within-ethnicity genetic principal components. We used the same method to incorporate each social/psychosocial factor separately into the model to test the interaction between the social/psychosocial factor and the SNP set within the risk gene/region on BMI after adjusting for the aforementioned covariates. The analyses were conducted separately for each ethnic ancestry and then were meta-analyzed using Fisher’s method [36]. We only formally tested gene-environment interactions for the social/psychosocial factors that were significantly associated with BMI in EA, AA, or in combined (EA + AA) meta-analysis. For each social/psychosocial factor, we applied false discovery rate (FDR) correction to the 97 gene/regions we tested. Last, we followed up significant gene-environment findings with single SNP-based analysis using GEE modeling for each of the SNPs within that gene/region to identify the SNPs that have the strongest interaction with the social/psychosocial factor.

### 2.6. Replication

MESA [37] is a longitudinal study of the characteristics of subclinical cardiovascular disease and the risk factors that predict progression to clinical cardiovascular disease. MESA began in 2002 with a sample of 6814 asymptomatic men and women aged 45–84 recruited in six communities: Baltimore, MD, USA; Chicago, IL, USA; Forsyth County, NC, USA; Los Angeles County, CA, USA; New York, NY, USA; and St. Paul, MN, USA. Approximately 38% of the participants are European American, 28% African American, 22% Hispanic, and 12% Asian. Five follow-up examinations and multiple ancillary studies have been conducted since MESA’s inception, and a sixth examination is currently underway. Data collected includes traditional coronary disease risk factors, socio-demographic factors, lifestyle factors, and psychosocial factors.

For this analysis, we used BMI collected at MESA Exams 1 through 4. Childhood SES was assessed analogously to the HRS using the maximum parental education of either parent. Genotype data was collected using Affymetrix Genome-wide Human SNP Array 6.0. Quality control was done prior to the imputation. In addition to the duplicated samples, samples with a large missing call rate (<95%) or gender mismatch were removed. SNPs were removed if they had call rates <95% or heterozygosity >53%. Genotyped data were imputed to the 1000 Genomes Project cosmopolitan reference panel phase 1 (version 3) using IMPUTE2 [30]. For the replication analyses, we included all high quality SNPs (INFO > 0.5 and MAF > 0.01) within the region of 50 kb around the index SNP rs9540493. After filtering, 248 SNPs in EA and 476 SNPs in AA were included in the analysis. The top 4 ethnic-specific principal components estimated using genome-wide SNPs data were used as covariates in all analyses. As with the HRS, we conducted gene/region-based analyses using LGEWIS and SNP-based analyses using GEE.

Both the HRS and MESA have been approved by Institutional Review Boards (IRBs) at the corresponding institutions, Study eResearch ID: HUM00119419. All participants provided informed consent. Collection and production of data comply with the IRB requirements. The analyses have been approved by the study-related Publication and Presentation committees.

## 3. Results

### 3.1. Descriptive Statistics

The descriptive statistics of BMI, the social/psychosocial factors, and covariates at Exam 1 and Exam 2 for the HRS are presented in Table 1.

### 3.2. Association between Social/Psychosocial Factors and BMI

GEE models were used to test the association between each social/psychosocial factor and BMI after controlling for age, age^2^ and sex. In EA, ASES, CSES, ANGEROUT, BURDEN, PSS, NSS, CESD, and SLE, were all significantly associated with BMI at a nominal level (*p* < 0.05) (Table 2). We applied Bonferroni correction to the nine social/psychosocial factors. The AESE, CSES, ANGEROUT, BURDEN, NSS, and SLE remained significant (*p* < 0.0056). The direction of the effect for all the significant social/psychosocial factors was consistent with the hypothesis that worse environments increase BMI. In AA, none of the social/psychosocial factors were significantly associated with BMI. Meta-analysis was conducted between EA and AA using inverse variance weighted fixed effect models. Five of the six social/psychosocial factors (ASES, CSES, ANGEROUT, BURDEN, and NSS) that were associated with BMI in EA remained significant in the meta-analysis after Bonferroni correction.

### 3.3. Association between Gene/Regions and BMI

Using LGEWIS, we tested the aggregated marginal effect of SNPs within each gene/region on BMI after controlling age, age^2^, sex and the top four principal components. By chance alone, we expect 5 genes with *p*-value < 0.05 and 10 genes with *p*-value < 0.1 in each ethnicity. In EA, 23 out of 97 genes were associated with BMI (*p* < 0.05), and 31 genes showed suggestive evidence of association with BMI (*p* < 0.1). This suggests that the 97 gene/regions are over-represented with BMI associated gene/regions in the EA sample. In AA, six genes were associated with BMI (*p* < 0.05) and 10 genes showed suggestive evidence of association with BMI (*p* < 0.1), which is likely by chance (Table S1). The gene that showed strongest association with BMI (*p* = 5.3 × 10^−5^) in EA was *FTO*, which was also the top gene identified through GWAS analysis for BMI.

### 3.4. Interaction between Genes and Social/Psychosocial Factors on BMI

In EA, we tested the interaction model for the environmental variables that were significantly associated with BMI (ASES, CSES, ANGEROUT, BURDEN, NSS, and SLE) and all 97 gene/regions. The strongest association was found between the gene/region containing index SNP rs9540493 and CSES (*p* = 0.000636). We applied FDR correction to the 97 gene/regions tested for each social/psychosocial factor. The interaction between the gene/region with index SNP rs9540493 and CSES was the only interaction that remained significant after FDR correction (*q* = 0.049). In AA, we did not formally test gene-environment interaction models because none of the social/psychosocial factors were associated with BMI.

Next, we performed meta-analysis between EA and AA gene-environment analysis results using Fisher’s method for the five social/psychosocial factors that were significantly associated with BMI in the EA + AA meta-analysis (ASES, CSES, ANGEROUT, BURDEN, and NSS). Following that, we applied FDR correction to the 97 meta *p*-values for each factor. The gene/region with index SNP rs9540493 and CSES had a significant interaction on BMI (*p_EA+AA_* = 0.000279, FDR *q*-value = 0.0216). Even though the interaction was significant in EA + AA meta-analysis, it was likely driven by EA. There was not strong evidence that the interaction was significant in AA alone, given that 97 gene/regions were tested (*p* = 0.038 before multiple testing correction).

**Figure 1:**
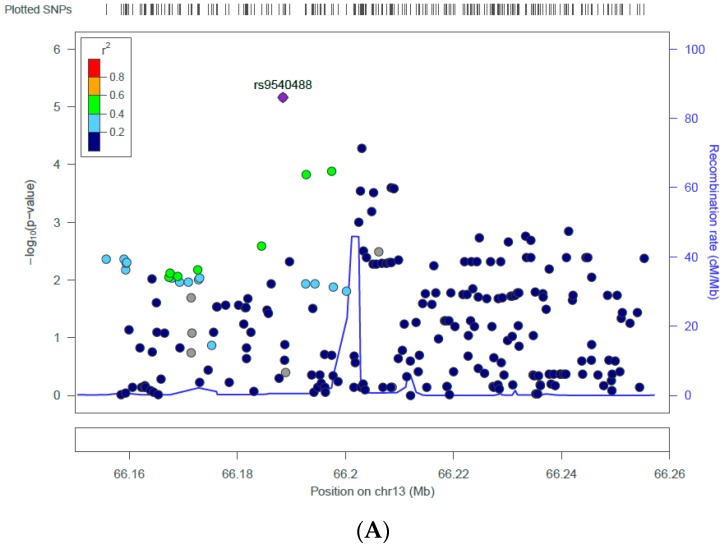

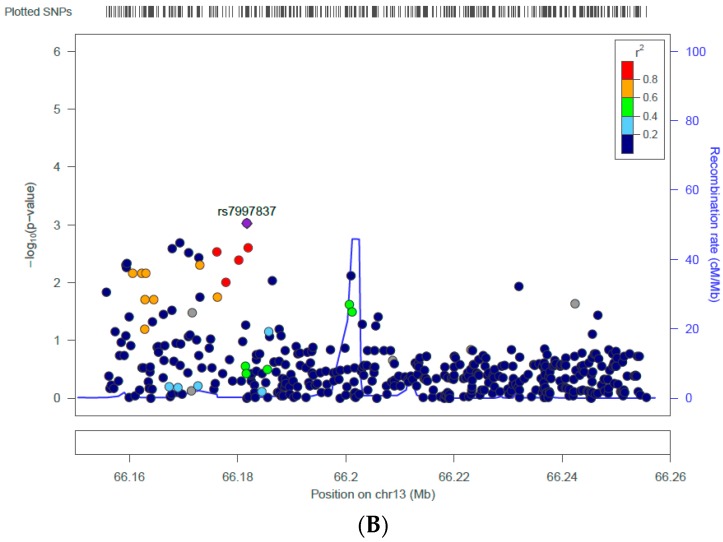
Zoom plot of the interaction p values (SNP, single nucleotide polymorphism by childhood socioeconomic status on BMI) for all the SNPs within the gene/region containing index SNP rs9540493 in European American (**A**) and African American (**B**) samples in the Health and Retirement Study (HRS). SNPs are plotted by chromosomal position (GRCh 37, x axis) against the –log (*p*-value) of their interaction with childhood socioeconomic status (parental education) on BMI (y axis). The most significant SNPs (rs9540488 in European Americans, rs7997837 in African Americans) are shown as purple diamonds. The SNPs surrounding the most significant SNPs are color-coded to reflect their linkage disequilibrium with this SNP as shown in the inset (taken from pairwise r^2^ values from the 1000 Genomes Project November 2014 release, EUR panel in A and AFR panel in B).

**Figure 2:**
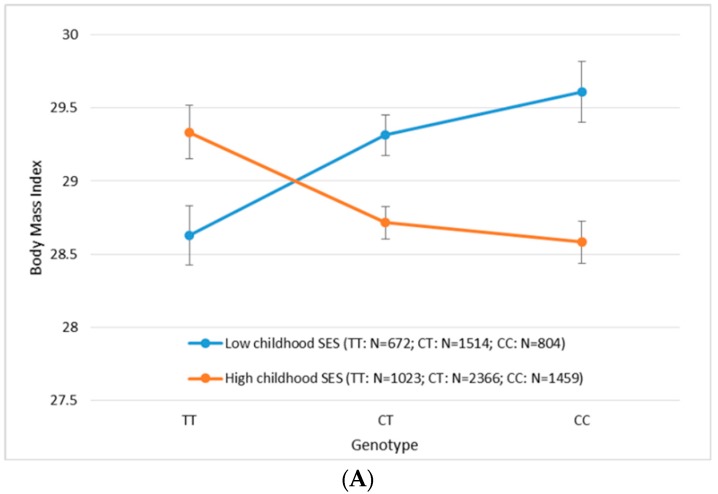

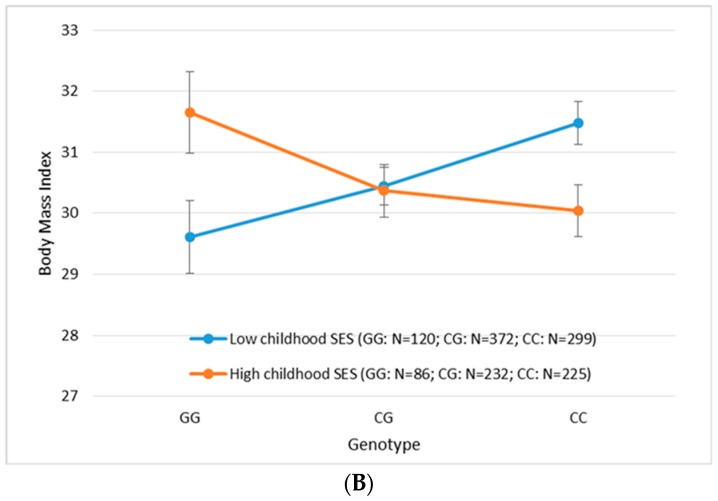
Interaction between the most significant SNPs and childhood socioeconomic status (SES measured by parental education) on BMI in the Health and Retirement Study (HRS). Average covariate-adjusted BMI value across multiple exams was calculated for each participant. Mean BMI was plotted for participants in high and low childhood SES groups against different genotypes of the SNP rs9540488 (*beta_snp_* = −0.38, *p_snp_* = 0.0013, *beta_parental education_* = −0.44, *p_parental education_* = 0.066, *beta_int_* = 0.85, *p_int_* = 6.91 × 10^−6^) in European Americans (**A**) and the SNP rs7997837 (*beta_snp_* = −0.67, *p_snp_* = 0.077, *beta_parental education_* = −1.70, *p_parental education_* = 0.019, *beta_int_* = 1.65, *p_int_* = 0.00095) in African Americans (**B**). Parameters were estimated from longitudinal analysis via GEE models separately for European American participants (*N* = 7838, *M* = 12,409) and African American participants (*N* = 1338, *M* = 1861). N: number of participants; M; number of observations.

Last, we conducted single SNP analysis for all of the SNPs within the rs9540493 gene/region in both the EA and AA samples. Similar to the gene-based analysis, we did longitudinal analysis incorporating repeated measures over time using GEE. The *p*-value of the interaction term (CSES by SNP) for each of the SNPs in each ethnic ancestry is shown in Figure 1 [38]. The most significant SNP in the EA sample was rs9540488 (*beta_int_* = 0.85, *p_int_* = 6.91 × 10^−6^), whereas the most significant SNP in the AA sample was rs7997837 (*beta_int_* = 1.65, *p_int_* = 0.00095). The plots in Figure 2 show how the genotypes of the most significant SNPs interact with the CSES to influence BMI. In both cases, for the participants with CC genotype for the corresponding top SNP, low CSES are associated with higher BMI. For the participants who are homozygous for the corresponding alternative allele, low CSES are associated with lower BMI. As a comparison, the top SNP (rs9540488) in EA did not have a significant interaction with CSES on BMI in the AA sample (*beta_snp_* = 0.24, *p_snp_* = 0.675, *beta_parental education_* = 0.22, *p_parental education_* = 0.878, *beta_int_* = 0.08, *p_int_* = 0.917). Likewise, the top SNP (rs7997837) in AA did not have a significant interaction with CSES on BMI in the EA sample (*beta_snp_* = −0.09, *p_snp_* = 0.807, *beta_parental education_* = 2.27, *p_parental education_* = 0.07, *beta_int_* = −0.92, *p_int_* = 0.149). The two top SNPs have different allele frequencies across ethnic ancestries. The frequency of C/T allele for rs9540493 are 0.538/0.462 in EA and 0.882/0.118 in AA, and the frequency of C/G allele for rs7997837 are 0.976/0.024 in EA and 0.621/0.379 in AA.

### 3.5. Replication Analyses in MESA

The descriptive statistics of key variables in MESA are presented in the Table S2. The mean age of MESA and HRS participants is similar (between 62 and 66 years of age for EA and AA at baseline), and they have similar average BMI (HRS EA = 29 kg/m^2^, AA = 31 kg/m^2^; MESA EA = 28 kg/m^2^, AA = 30 kg/m^2^). Compared to the HRS, MESA participants had a smaller proportion of women and smaller proportion of participants who are in the low CSES category. Using MESA data, we sought to replicate our four top findings: gene/region rs9540493 by CSES interaction on BMI in EA; gene/region rs9540493 by CSES interaction on BMI in EA + AA; SNP rs9540488 by CSES interaction on BMI in EA; and rs7997837 by CSES interaction on BMI in AA. For gene-based analysis, the interaction between gene/region rs9540493 and CSES on BMI was not significant in EA (*p* = 0.17) or EA + AA (*p* = 0.18). When the top SNPs were examined individually in the corresponding ethnic groups, the interaction between rs9540488 and CSES in EA was suggestively significant (*p* = 0.071), with consistent directions of effect in both cohorts. For people with TT genotypes, low CSES decrease BMI. For people with CC genotypes, low CSES increase BMI. For people with CT genotypes, CSES does not have a strong effect. However, the interaction between rs7997837 and CSES in AA was not significant (*p* = 0.427). The test statistics for the rs9540488 by CSES interaction in EA in both MESA and the HRS are presented in Table 3. The effect directions are consistent between the two studies, and the effect sizes are similar as well. The SE is much larger in MESA compared to the HRS due to small sample size, which leads to larger p value than we observed in the HRS. These results show that the interaction between rs9540499 and CSES in EA was suggestively replicated in MESA.

## 4. Discussion

This is the first study to apply gene/region-based methods to examine how social/psychosocial factors modify genetic effects on BMI across multiple ethnic groups in large population-based studies. Given the longitudinal study design and the heterogeneity of the genetic effects across ethnicity, we applied a novel gene/region-based method for longitudinal studies, LGEWIS, to characterize the overall effect of gene/region by environmental interaction on BMI in older adults from the HRS. Childhood socioeconomic status, as measured by parental education, was found to modify the genetic effect in the rs9540493 gene/region on BMI in EA and in combined analysis with AA. The effect was suggestively replicated in MESA EA.

It has been noted that low SES is associated with being overweight and obesity [39,40]. This relationship may be mediated by several pathways. For example, nutrition and exercise, which are largely determined by personal lifestyle choices such as fast food consumption, skipping breakfast, and physical inactivity, which are all strongly associated with lower SES as well as higher BMI [41,42]. From a developmental point of view, childhood is considered a critical period that can have profound effects on many aspects of health and disease in later life. Many of the aforementioned life style choices may have been shaped during childhood. Indeed, low childhood SES has been found to be associated with higher BMI and some obesity related traits in adulthood [43,44,45,46,47]. Childhood socioeconomic status can be measured in a variety of ways including, but not limited to, parental education, parents’ occupation, household income, and wealth [48,49]. In this study, we chose parental education because it is stable over the life course and strongly predicts occupation and income [50,51]. Among a variety of other SES indicators, fewer years of education of the parents was found to be the factor most strongly negatively associated with childhood obesity [52].

In addition to environmental factors, obesity also has a strong genetic component. Heritability estimates of BMI range from 40% to 70% [6,7]. But SNPs identified through GWAS only explain a small proportion of the variance of BMI. This missing heritability could be due to gene-environment interactions [53,54,55]. However, it is difficult to detect gene-environment interactions evaluated on a genome-wide scale due to the massive sample sizes needed to detect interaction effects. The few studies that have examined interaction between genetics and the psychosocial environment interaction have focused only on a few key SNPs from one or two genes [56]. The present study is the first study to our knowledge to survey a variety of social/psychosocial factors and more than 113K SNPs from 97 genes/regions. CSES was found to modify the genetic effect of the rs9540493 gene/region on BMI in EA and in the combined EA + AA sample. Additionally, this gene/region was nominally significant (*p* = 0.038) in AA suggesting that the gene/region may have a similar effect across ethnicity. When the interaction between CSES and individual SNPs within the gene/region were closely examined, the most significant SNPs were different between EA and AA. This is not surprising due to known heterogeneity in allele frequencies and linkage disequilibrium (LD) patterns across different ancestry groups. Indeed, the lead SNPs in both the EA and AA samples have very different allele frequency across ethnic ancestries and they are not in LD with each other. This reinforces that the gene-based approach may be valuable for trans-ethnic analysis. More importantly, the LGEWIS method allows incorporation of unbalanced longitudinal data, which can capture the time varying component of traits such as BMI. Following the discovery analyses, we sought to replicate our top findings in MESA, an independent cohort. Only the top SNP (rs9540488) in EA was suggestively replicated in MESA EA, which provides further evidence for the interaction between CSES and this gene region. However, we did not replicate this finding in MESA AA, suggesting that the interaction may only be present in EA. In terms of the effect size, we compared our observed effect sizes in the HRS to the effect sizes from the largest GWAS of BMI to date [5]. In Locke et al. (2015), the analysis was done using the inverse normalized residual of BMI (after adjusting all the covariates) as the outcome. Thus, we reanalyzed the SNP (rs9540488) by CSES interaction in a similar way in the HRS. The beta estimate of the SNP and of the SNP by CSES interaction were −0.068 and 0.143 respectively. This suggests the SNP rs9540488 has a heterogeneous effect in different CSES categories with beta estimates of −0.068 and 0.075 for participants in low and high CSES categories. These effect sizes are comparable to the effect sizes reported by Locke et al. (0.017 to 0.082). We also tested for the association between the SNP and BMI without considering CSES, analogous to the analysis performed by Locke, et al. The beta estimate for the SNP was −0.017 and not significant. This indicates that we observed larger effects within homogeneous CSES categories than across heterogeneous CSES categories. As illustrated by this analysis, GWAS analyses may not identify SNPs that have heterogeneous effects across environmental categories if non-genetic environments are not considered. Thus, investigating gene-environment interaction could be a very important next step in genetic analyses.

We selected gene/regions for evaluation based on index SNPs from the largest GWAS of BMI to date [5]. In that study, two genes, *MIR548X2* and *PCDH9*, were noted to be in the region of the index SNP rs9540493. *MIR548X2* is the nearest gene to rs9540493 and is a short non-coding RNA. It is known to regulate post transcriptional gene expression in multiple cells and tissues [57]. It is not clear how this gene is related to BMI. Gene *PCDH9* interacts with calcium to mediate cell adhesion in the central nervous system and may also be involved in neural transmission [57]. It was predicted to be the most possible BMI regulating gene in this region by DEPICT [58] due to the fact that there is strong evidence that the discovered BMI risk genetic locus are enriched or highly expressed or known to have an effect in the brain and central nervous system [5,59]. The fact that the gene was expressed in different brain areas at different time points post fertilization suggests that it plays a role in morphogenesis [60,61]. This developmentally regulated expression pattern could create a critical period, like early childhood, which is more sensitive to the modification effect of environment factors. In addition to the central nervous system, the gene is also expressed in a variety of other tissues. Functional experiments are needed to validate the effect of the gene. Another interesting fact is a SNP (rs1333026) located 80kb upstream of rs9540493 was also found to be associated with BMI in an earlier study [62]. A fine mapping study in the expanded region might be needed to identify the causal variants and reveal the underlying mechanism.

In this study, we also examined the association between a variety of social/psychosocial factors and BMI in the EA and AA samples in the HRS. Out of the nine social/psychosocial factors, six (ASES, CSES, ANGEROUT, BURDEN, NSS, and SLE) were significantly associated with BMI in the EA sample. For these results, the direction of the effect is consistent with our hypothesis that adverse social/psychosocial environment increases BMI. None of these relationships were significant in African Americans, which may be due to the small sample size. Another reason may be the heterogeneity in the social/psychosocial measurements. Many of the environmental factors we examined were psychological factors, which are measured by questionnaire. The measurement is subjective, and different populations may have different sensitivity levels towards the questionnaire or differing interpretations of the questions [63]. Even for the more objective measures, like education, the same education level may not place people from different ethnic ancestries in the same relative socioeconomic status. It has been observed that educational attainment does not offer the same health benefit for AA as EA [64]. For parental education, we observed the same inverse relationship between parental education and BMI in EA and AA. Even though this relationship was not significant in AA, the direction of the effect and the effect size were very similar to EA.

As a confirmatory analysis, we also conducted gene-based analysis to examine the marginal genetic effect of the selected gene/regions on BMI. Out of the 97 gene/regions examined, 31 in EA and 10 gene/regions in AA were marginally associated with BMI. More importantly, the most significant gene/region was the *FTO* gene, which was the most significant gene discovered through GWAS as well [4,5,65,66]. This supports the validity of the gene/regions we selected for analysis, as well as our analytic approach. Of note is that the GWAS signals were discovered largely through analyses conducted on European ancestry [5]. Concern might be raised about the heterogeneity of the SNP effects across different ancestries. It is true that we do not expect the most significant SNPs discovered in European ancestry to have similar effects in African ancestry. Genes, however, are expected to have similar function across ethnic ancestries. Our approach, either selecting an entire gene or selecting 50kb around the index SNP, was likely to have successfully captured the functional genetic unit or complete LD block of the region of interest [67]. The observation that we were only able to replicate a genetic effect on BMI in a few genes in AA is more likely due to the small sample size than differences in the underlying genetic influences on BMI across ethnic ancestries.

## 5. Conclusions

In summary, by using the novel method LGEWIS, we identified a gene/region around SNP rs9540493 whose genetic effect on BMI was modified by childhood SES in participants of European ancestry. We anticipate this novel work to be particularly important in illustrating a working model for examining gene-environment interaction. A greater understanding of gene-environment interaction on BMI will increase knowledge of the etiology of obesity and potentially provide guidance for designing more effective strategies for reducing BMI in target populations.

## Figures and Tables

**Table 1:** Descriptive statistics of the social/psychosocial factors and covariates in the Health and Retirement Study (HRS).

Variable Name	European Americans (*N* = 7838)		African Americans (*N* = 1334)	
	Exam 1 (*M* = 7838)	Exam 2 (*M* = 5746)	Exam 1 (*M* = 1334)	Exam 2 (*M* = 527)
Outcome				
BMI, Mean (SD)	29 (5.7)	29 (5.5)	31 (6.5)	30(6.3)
Demographics				
Age, Mean (SD)	66 (10)	70 (9)	63 (9.9)	69 (8.8)
Sex (Female), *N* (%)	4447 (56.7)	2618 (57.3)	863 (64.7)	356 (67.6)
Social Factors				
ASES (low), *N* (%)	3790 (48.4)	2145 (46.9)	790 (59.2)	315 (59.8)
CSES (low), *N* (%)	2990 (38.1)	1724 (37.7)	791 (59.3)	338 (64.1)
Psychosocial Factors				
ANGERIN (high), *N* (%)	5414 (69.1)	3035 (66.4)	864 (64.7)	317 (60.2)
ANGEROUT (high), *N* (%)	3201 (40.8)	1666 (36.4)	626 (46.9)	224 (42.5)
BURDEN * (high), *N* (%)	2751 (57.4)	2520 (55.4)	504 (56.3)	269 (51.9)
PSS (low), *N* (%)	4000 (51.0)	2343 (51.3)	669 (50.2)	259 (49.2)
NSS (low), *N* (%)	4126 (52.6)	2174 (47.6)	696 (52.2)	237 (45.0)
CESD (high), *N* (%)	4066 (51.9)	2560 (56.0)	460 (34.5)	222 (42.1)
SLE (high), *N* (%)	1587 (20.2)	912 (20.0)	395 (29.6)	140 (26.6)

* Sample size for BURDEN: *M* = 4789 at Exam 1 and *M* = 4552 at Exam 2 in European Americans; *M* = 895 at Exam 1 and *M* = 518 at Exam 2 in African Americans. *N* = number of individuals; *M* = number of observations. ASES (low): adult socioeconomic status, (less than or equal to 12 years of education); CSES (low): childhood socioeconomic status (less than high school degree for the highest educational attainment of either parent); ANGERIN (high): trait anger score (higher than the ethnicity-specific median); ANGEROUT (high): state anger score (higher than the ethnicity-specific median); BURDEN (high): chronic burden score (higher than the ethnicity-specific median); PSS (low): positive social support score (lower than the ethnicity-specific median); NSS (low): negative social support score(lower than the ethnicity-specific median); CESD (high): depression score (higher than 0); SLE (high): stressful life events score (higher than 0). All of the above social/psychosocial categories were coded as 1 (reference is 0) in the analysis.

**Table 2:** Association between social/psychosocial factors and body mass index (BMI) from longitudinal analysis using generalized estimation equation models in the Health and Retirement Study (HRS).

Social/Psychosocial Factors	European Americans (*N* = 7838)			African Americans (*N* = 1334)			Meta-Analysis		
	Beta	SE	*p*-Value	Beta	SE	*p*-Value	Beta	SE	*p*-Value
Social Factors									
ASES (low)	0.83	0.127	**3.69 × 10^−11^**	0.40	0.353	0.2585	0.79	0.119	**3.87 × 10^−11^**
CSES (low)	0.48	0.134	**0.0004**	0.40	0.375	0.2812	0.47	0.126	**0.0002**
Psychosocial Factors									
ANGERIN (high)	0.10	0.059	0.0898	−0.27	0.204	0.1822	0.07	0.057	0.21
ANGEROUT (high)	0.25	0.062	**4.41 × 10^−5^**	−0.07	0.198	0.7208	0.23	0.059	**0.0002**
BURDEN * (high)	0.39	0.076	**2.46 × 10^−7^**	0.20	0.232	0.3988	0.37	0.072	**2.37 × 10^−7^**
PSS (low)	0.14	0.064	0.0254	−0.08	0.172	0.6408	0.12	0.060	0.0533
NSS (low)	0.20	0.063	**0.0014**	0.17	0.182	0.3639	0.20	0.060	**0.0009**
CESD (high)	−0.13	0.058	0.0221	0.07	0.188	0.7195	−0.12	0.056	0.0376
SLE (high)	0.20	0.072	**0.0044**	−0.23	0.206	0.2549	0.16	0.068	0.0205

* Sample size for BURDEN: *N* = 4789 in European Americans and *N* = 895 in African Americans. ASES (low): adult socioeconomic status, (less than or equal to 12 years of education); CSES (low): childhood socioeconomic status (less than high school degree for the highest educational attainment of either parent); ANGERIN (high): trait anger score (higher than the ethnicity-specific median); ANGEROUT (high): state anger score (higher than the ethnicity-specific median); BURDEN (high): chronic burden score (higher than the ethnicity-specific median); PSS (low): positive social support score (lower than the ethnicity-specific median); NSS (low): Negative social support score(lower than the ethnicity-specific median); CESD (high): depression score (higher than 0); SLE (high): stressful life events score (higher than 0). All the above social/psychosocial categories were coded as 1 (reference is 0) in the analysis. Beta represents the mean difference of BMI value between the two categories of each social/psychosocial factors. Significant *p*-values are bolded (Bonferroni corrected significance threshold is 0.0056).

**Table 3:** Interaction between SNP rs9540488 and childhood socioeconomic status in European Americans (EA) in the Health and Retirement Study (HRS) and the Multi-Ethnic Study of Atherosclerosis (MESA).

Effect	HRS EA (*N* = 7838)			MESA EA (*N* = 2366)		
	Beta	SE	*p*-Value	Beta	SE	*p*-Value
rs9540488 (coded allele C)	−0.38	0.118	0.0013	−0.23	0.182	0.207
CSES (low)	−0.44	0.239	0.066	−0.186	0.413	0.653
rs9540488 * CSES (low)	0.85	0.190	6.90 × 10^−6^	0.582	0.322	0.071

* CSES (low): childhood socioeconomic status (less than high school degree for the highest educational attainment of either parent). Beta represents the change in BMI (kg/m^2^) associated with different genotypes and/or CSES categories.

## Supplementary Materials

The following are available online at www.mdpi.com/1660-4601/14/10/1153/s1, Methods for genotyping and imputation in the Health and Retirement Study (HRS), Table S1: The marginal effect of the gene/regions on BMI in European Americans and African Americans in the Health and Retirement Study (HRS), Table S2: Descriptive statistics of the outcome and covariates in the Multi-Ethnic Study of Atherosclerosis (MESA).
