# Supplementary material for: Interaction between Social/Psychosocial Factors and Genetic Variants on Body Mass Index: A Gene-Environment Interaction Analysis in a Longitudinal Setting
Source: Int J Environ Res Public Health. 2017 Sep 29;14(10):1153. doi: 10.3390/ijerph14101153 (PMC5664654; doi:10.3390/ijerph14101153)
Supplement: Supplementary file 1 [file ijerph-14-01153-s001.pdf]

International Journal of
Environmental Research and Public Health

MDPI

Supplementary Material

Interaction between Social/Psychosocial Factors and Genetic Variants on Body Mass Index: A Gene-Environment Interaction Analysis in a Longitudinal Setting

Wei Zhao, Erin B. Ware, Zihuai He, Sharon L.R. Kardia, Jessica D. Faul and Jennifer A. Smith

Methods for genotyping and imputation in the Health and Retirement Study (HRS)

Genotyping was conducted by the Center for Inherited Disease Research (CIDR). Genotype data was obtained using the Illumina HumanOmni2.5 BeadChip, which measures ~2.4 million single nucleotide polymorphisms (SNPs). Individuals a with call rate <98% and with first degree relatives in the HRS were excluded from analysis. Principal components analysis was used to calculate genetic principal components (PCs) using SNPRelate [1]. The top two PCs and self-reported ethnic ancestry were used to select an analysis sample of unrelated European American (EA, $N = 9991$) and African American (AA, $N = 2279$) participants. SNPs a with call rate <98%, ethnicity-specific Hardy Weinberg equilibrium (HWE) p-value < 0.0001, and those in regions with chromosomal anomalies were also excluded. Imputation to the 1000 Genomes Project cosmopolitan reference panel phase 1, version 3 (released on March 2012) was performed using SHAPEIT2 [2] and IMPUTE2 [3]. Overall, ~23 million SNPs were imputed from the original 2,118,384 SNPs that were genotyped and passed quality control. Masking of genotyped SNPs to assess the accuracy of imputation was performed to estimate the median concordance between actual and imputed genotypes. The mean of empirical dosage correlation r^2 was 0.817 for rare variants (MAF < 0.05) and 0.953 for common variants (MAF ≥ 0.05), and additional quality control metrics indicate high quality imputation. For each ethnicity, genetic PCs were generated from common SNPs (MAF > 0.05) and were used as covariates to adjust for population stratification.

Table S1. The marginal effect of the gene/regions on BMI (Body Mass Index) in European Americans and African Americans in the Health and Retirement Study (HRS).

Gene/Region	European Americans		African Americans	
	N markers	*P* value	*N* markers	*P* value
AGBL4	2483	0.220	4236	0.657
ASB4	244	0.337	366	0.206
BDNF	446	0.128	707	0.762
C18orf8	209	**0.023**	493	0.242
C6orf106	321	**0.017**	601	0.936
CADM2	3525	0.373	6132	0.420
CALCR	480	0.285	841	0.147
CCDC171	1756	0.162	3322	0.578
CLIP1	341	0.077	811	0.716
DMXL2	500	0.158	823	0.879
DNAJB4	141	0.506	182	0.584
EHBP1	499	0.898	764	0.785
ELAVL4	227	0.073	408	0.136
ETV5	144	**0.045**	229	0.500
FHIT	7287	0.116	11583	0.194
FIGN	341	0.673	624	0.446
FOXO3	237	0.647	493	0.069
FTO	1513	$\mathbf{5.3 \times 10^{-5}}$	2394	0.460

GBE1	645	**0.042**	1126	0.711
GNAT2	38	0.326	71	0.827
GRID1	2598	**0.031**	4259	0.917
HHIP	234	0.446	423	0.651
HIP1	854	**0.014**	1242	0.597
HSD17B12	833	0.113	1327	0.265
INO80E	41	**0.004**	75	0.788
KAT8	47	0.883	92	0.730
KCNK3	123	0.179	240	**0.0496**
LINC00907	1843	0.559	3249	0.585
LINGO2	2998	0.119	5139	**0.045**
LMX1B	344	**0.014**	545	0.791
MAP2K5	742	**0.036**	1166	0.150
MTCH2	44	**0.027**	112	0.848
NAV1	583	0.302	1185	0.737
NLRC3	131	0.051	300	0.615
NRXN3	4580	0.421	8562	0.368
NT5C2	334	0.458	417	0.105
PARK2	5966	0.364	10157	0.102
PGPEP1	140	**0.0498**	232	0.365
PRKD1	1794	0.316	3226	0.136
QPCTL	60	0.223	102	**0.034**
RABEP1	472	0.051	642	0.942
RASA2	241	0.260	632	0.619
RPTOR	2037	0.526	3180	0.469
rs10132280	375	0.096	607	0.538
rs1016287	316	0.766	538	0.492
rs10182181	353	**0.002**	505	0.481
rs10938397	317	0.155	655	0.774
rs11165643	357	0.201	542	0.234
rs12016871	441	0.870	744	0.873
rs12286929	294	0.239	553	0.922
rs12429545	266	0.142	477	0.871
rs12446632	258	0.109	531	0.609
rs12885454	227	0.247	437	0.640
rs13021737	599	0.112	925	0.205
rs13201877	343	0.779	562	0.191
rs1441264	329	0.566	597	0.052
rs1528435	376	0.189	505	0.097
rs16907751	316	0.872	494	0.595
rs17094222	263	0.144	526	0.726
rs17203016	294	0.640	472	0.740
rs17405819	289	$\mathbf{4.6 \times 10^{-4}}$	428	0.581
rs1928295	354	0.401	442	0.205
rs2033529	439	**0.002**	700	0.125
rs2033732	267	**0.018**	481	0.347
rs2080454	287	0.183	570	0.587
rs2112347	364	**0.015**	610	0.942
rs2121279	279	0.574	451	0.296
rs2176040	188	**0.009**	540	**0.026**
rs2207139	290	0.594	434	**0.016**
rs2245368	248	0.096	320	0.761
rs2836754	412	0.767	645	0.856
rs29941	400	0.863	605	0.428
rs3101336	265	0.469	561	0.681
rs3888190	219	0.064	361	0.254
rs543874	376	**0.033**	523	0.476

rs6091540	255	0.762	374	0.863
rs6477694	437	0.197	571	0.431
rs6567160	244	**0.025**	527	0.354
rs6804842	418	0.619	763	0.095
rs7138803	361	**0.035**	586	0.801
rs7164727	227	**0.043**	418	**0.033**
rs7243357	517	0.252	794	0.838
rs7599312	341	0.143	541	0.201
rs7715256	334	0.111	459	0.762
rs9374842	400	0.319	478	0.628
rs9540493	261	0.343	478	0.769
SBK1	76	0.389	131	0.347
SCARB2	253	0.198	509	0.444
SLC39A8	706	0.921	1126	0.439
SMG6	756	0.456	1060	0.156
TAL1	62	0.942	132	0.699
TCF7L2	573	0.330	972	0.394
TNNI3K	801	0.682	1908	0.158
TOMM40	72	**0.009**	123	0.267
TRIM66	177	0.731	292	0.404
USP37	276	0.562	540	0.732
ZC3H4	110	0.092	271	0.644

LGEWIS was used to test the association between gene/regions and BMI adjusting for age, sex and the top four genetic principal components. The tests were done separately for the European American and African American samples. *P*-values < 0.05 are in bold. "N markers" refers to the number of SNPs for each gene/region that was included in analysis.

Table S2. Descriptive statistics of the outcome and covariates in the Multi-Ethnic Study of Atherosclerosis (MESA).

**European American (N=2366)**				
**Variable Name**	**Exam 1 (*M* = 2359)**	**Exam 2 (*M* = 2361)**	**Exam 3 (*M* = 2250)**	**Exam 4 (*M* = 2165)**
Age, mean (SD)	63 (10.2)	64 (10.2)	65 (10.0)	67 (10.0)
BMI, mean (SD)	28 (4.9)	28 (4.9)	28 (5.0)	28 (5.1)
Gender (Female), N (%)	1211 (51.3)	1212 (51.3)	1157 (51.4)	1112 (51.4)
CSES (low), N (%)	677 (28.7)	679 (28.8)	630 (28.0)	603 (27.9)
**African American (*N* = 1413)**				
**Variable Name**	**Exam 1 (*M* = 1412)**	**Exam 2 (*M* = 1409)**	**Exam 3 (*M* = 1318)**	**Exam 4 (*M* = 1257)**
Age, mean (SD)	62 (10.0)	64 (9.9)	65 (9.8)	66 (9.6)
BMI, mean (SD)	30 (5.7)	30 (5.7)	30 (5.7)	30 (5.8)
Gender (Female), N (%)	746 (52.8)	744 (52.8)	703 (53.3)	669 (53.2)
CSES (low), N (%)	623 (44.1)	623 (44.2)	581 (44.1)	554 (44.1)

CSES (low): childhood socioeconomic status (below high school degree for the highest educational attainment of either parent); N: number of individuals: M: number of observations.

References

1. Zheng, X.; Levine, D.; Shen, J.; Gogarten, S.M.; Laurie, C.; Weir, B.S. A High-Performance Computing Toolset for Relatedness and Principal Component Analysis of SNP Data. *Bioinformatics* **2012**, *28*, 3326–3328.
2. Delaneau, O.; Zagury, J.F.; Marchini, J. Improved Whole-Chromosome Phasing for Disease and Population Genetic Studies. *Nat. Methods* **2013**, *10*, 5–6.
3. Howie, B.; Fuchsberger, C.; Stephens, M.; Marchini, J.; Abecasis, G.R. Fast and Accurate Genotype Imputation in Genome-Wide Association Studies through Pre-Phasing. *Nat. Genet.* **2012**, *44*, 955–959.
